# A review of the Neotropical genus *Bidessodes* Régimbart, 1895 including description of four new species (Coleoptera, Adephaga, Dytiscidae, Hydroporinae, Bidessini)

**DOI:** 10.3897/zookeys.658.10928

**Published:** 2017-02-22

**Authors:** Kelly B. Miller

**Affiliations:** 1 Department of Biology and Museum of Southwestern Biology, University of New Mexico, Albuquerque, NM 87131-0001 USA

**Keywords:** Water beetles, taxonomy, revision, Neotropical, *Bidessodes*, Dytiscidae

## Abstract

The Neotropical genus *Bidessodes* Régimbart, 1895 is reviewed. Four new species are described, *Bidessodes chlorus* Miller, **sp. n.**, *Bidessodes erythros* Miller, **sp. n.**, *Bidessodes leukus* Miller, **sp. n.**, and *Bidessodes melas* Miller, **sp. n.**, bringing the total number of species in the genus to 20. A key to species is provided. Important diagnostic features are illustrated and described and distributions of all species based on examined specimens and published records are provided. Recognition of the subgenera of *Bidessodes* is not justified, and the two names *Hughbosdineus* Spangler, 1981 **syn. n.** and *Youngulus* Spangler, 1981 **syn. n.**, described at the genus rank, are placed in synonymy with *Bidessodes*.

## Abstract (es)

El género neotropical *Bidessodes* Régimbart, 1895 se revisa. Cuatro nuevas especies se describen, *Bidessodes chlorus* Miller, **sp. n.**, *Bidessodes erythros* Miller, **sp. n.**, *Bidessodes leukus* Miller, **sp. n.**, y *Bidessodes melas* Miller, **sp**. **n.**, con lo que el número total de especies en el género se eleva a 20. Se proporciona una clave para las especies. Características diagnósticas importantes se ilustran y describen y se proporcionan las distribuciones de todas las especies basándose en los especímenes examinados y registros publicados. El reconocimiento de los subgéneros de *Bidessodes* no está justificado, y los nombres *Hughbosdineus* Spangler, 1981 **syn. n.** y *Youngulus* Spangler, 1981 **syn. n.**, descritos como géneros, son sinonimizados con *Bidessodes*.

## Introduction

The Neotropical genus *Bidessodes* Régimbart, 1895 currently includes 16 described species (Nilsson 2016) distributed among three subgenera. Thirteen are in the nominal subgenus with two in Bidessodes (Hughbosdinius) Spangler, 1981 and one in Bidessodes (Youngulus) Spangler, 1981. Historically, a group of similar looking species of Bidessini in Australia were also placed in this genus, but these were transferred to *Neobidessodes* Hendrich and Balke thereafter restricting *Bidessodes* to the New World (Hendrich et al. 2009). Relationships of *Bidessodes* to other Bidessini genera are not yet clear. The New World *Bidessodes* were revised by Young (1986).

Within Bidessini, *Bidessodes* is very similar to *Neobidessodes*, but *Bidessodes* have a series of fine denticles along the posterior margins of abdominal ventrites III–V that are absent in *Neobidessodes*. Several species have conspicuous male dimorphisms, particularly in the meso- and metalegs, prosternum and prosternal process, and last abdominal ventrite. The male genitalia (both the median and lateral lobes) are usually strikingly complex, and the male median lobe is bilaterally symmetrical and deeply bifid.

The first *Bidessodes* species were described in *Bidessus*, but most were later described in *Bidessodes*. Three of these species were described in the genera *Youngulus* Spangler, 1981 and *Hughbosdineus* Spangler, 1981, based especially on unusual modifications of males (Spangler 1981). These genera were soon placed as subgenera of *Bidessodes* by Young (1986).

The genus includes a mix of species some of which are relatively abundant and widespread, and others that are rare and restricted in distribution. They occur in shallow lentic and lotic (especially sandy forest stream) habitats.

The purpose of this research is to describe four new species discovered in northern South America as the result of focused collecting in the region. Most *Bidessodes* species are very distinctive, and new species are relatively easily recognized and diagnosed from others. Because a number of species were described since the last revision (Braga and Ferreira-Jr. 2009) and there have been nomenclatural changes (Hájek 2012; Hendrich et al. 2009), the entire genus is briefly reviewed here.

## Material and methods

**Measurements.** Measurements were made with an ocular scale on a Zeiss Discovery V8 dissecting microscope. The diagnostic range of measurements of structures was emphasized, so the largest and smallest specimens were preferentially measured. Measurements include: 1) total length (TL), 2) greatest width across elytra (EW), 3) greatest width of pronotum (PW), 4) greatest width of head (HW), and 5) distance between eyes (ED). The ratios TL/EW and HW/ED were also calculated.

**Images.** Illustrations were made using a drawing tube on a Zeiss Discovery V8 dissecting scope. Sketches were first done in pencil then scanned, placed into an Adobe Illustrator artboard and “inked” digitally using vector lines.

**Material.** Specimens of all species were examined except *Bidessodes fragilis* Régimbart, 1900, the identity of which is in question since the type is a female (Young 1986), and female specimens are difficult to distinguish. Type specimens were not examined, but there seems to be little question about the identity of any species in the group except *Bidessodes fragilis* (Young 1986). Specimens were examined from the following collections:

**CSBD** Center for Biological Diversity, University of Guyana (type specimens currently reposed with KUNHM)

**FSCA** Florida State Collection of Arthropods, University of Florida, Gainesville, FL, USA (P. Skelley)

**KBMC** Kelly B. Miller Collection, Museum of Southwestern Biology, University of New Mexico, Albuquerque, NM, USA

**KUNHM** University of Kansas Natural History Museum, University of Kansas, Lawrence, Kansas, USA (A.E.Z. Short)

**MIZA** Museo del Instituto de Zoología Agrícola Francisco Fernández Yépez, Universidad Central de Venezuela, Maracay, Venezuela (L. Joly)

**MSBA** Museum of Southwestern Biology Division of Arthropods, University of New Mexico, Albuquerque, NM, USA (K.B. Miller)

**NZCS** National Zoological Collection of Suriname, Paramaribo, Suriname (P. Ouboter)

**USNM** United States National Collection of Insects, Smithsonian Institution, Washington, DC, USA (T. Erwin)

**Distribution maps.** Dot maps presented here are derived from examined specimens and specific localities reported by Spangler (1981), Young (1986) and Braga and Ferreira-Jr. (2009).

### Taxonomic characters

*Head*. The anterior clypeal margin is vaguely thickened in many *Bidessodes*, such as *Bidessodes semistriatus*, but this is usually indistinct and not especially useful as a taxonomic character. Punctation of the head has been used historically as a taxonomic character, also, with some species with very sparse punctation and others with it more distinctive (Zimmermann 1921). Reassessment of this character suggests that it is also ambiguous at best.

*Pronotum*. The pronotum ranges from laterally strongly curved to nearly parallel-sided. The pronotal striae (plicae) are somewhat variable in length between species but extend usually about 1/3 the distance across the pronotum.

*Elytra*. The coloration of the elytra is variable between species with some nearly immaculate, others vaguely or indistinctly maculate and others more distinctly maculate or longitudinally striate.

*Prosternum*. The prosternum in some species is medially longitudinally carinate and setose in either just males (*Bidessodes obscuripennis* (Zimmermann, 1921)) or in both males and females (*Bidessodes knischi* (Zimmermann, 1921)). Most species have the prosternum and prosternal process not conspicuously modified. The prosternal process is somewhat variable in shape, however, with lateral margins curved to subparallel and the apex truncate to broadly rounded to pointed or acuminate. The process may be longitudinally grooved, slightly convex or flat.

*Metasternum*. There is a distinctive transverse impression across the metaventrite in *Bidessodes knischi*. Other species are unmodified.

*Legs*. The male pro- and mesotarsomeres are generally more or less broadly expanded than those of the female as occurs in most species of Dytiscidae. This is less evident in some species, especially *Bidessodes subsignatus* (Zimmermann, 1921). In some species, the male mesofemur is apically swollen or expanded. In several species, the base of the male mesotibia is distinctly bent (e.g. Fig. 66). A few species have the metatibia and metafemur expanded or otherwise modified in characteristic ways (e.g. Figs 50, 81).

*Male genitalia*. The male genitalia hold the best set of diagnostic features for species identification. The median lobe in all species is bilaterally symmetrical but is highly species-specific in shape. The lateral lobes are bisegmented and bilaterally symmetrical, as are most Bidessini, and their shapes are highly species-specific, similar to the median lobe. Some are quite complex in shape.

## Taxonomy

### Bidessodes

Taxon classificationAnimaliaORDOFAMILIA

Régimbart, 1895

Bidessodes Régimbart, 1895:76; type species: Bidessodes elongatus Sharp, 1882b:25 by monotypy.
Bidessodes Régimbart, 1900:528; type species: Bidessodes semistriatus Régimbart, 1900:529 by subsequent designation of Young 1969:2; preoccupied by Régimbart 1895:76; Blackwelder 1944:76; Young 1967:82; 1969:2; 1986:219; Biström 1988:7; Nilsson 2016:98.
Bidessus (Bidessodes) , Zimmermann, 1919:61; 1921:200.
Hughbosdineus Spangler, 1981:65 **syn. n.**
Youngulus Spangler, 1981:69 **syn. n.**
Bidessodes (Hughbosdineus) , Young, 1986:206; Biström, 1988:7.
Bidessodes (Youngulus) , Young, 1986:207; Biström, 1988:7.

#### Diagnosis.

**Figures 1–20.**
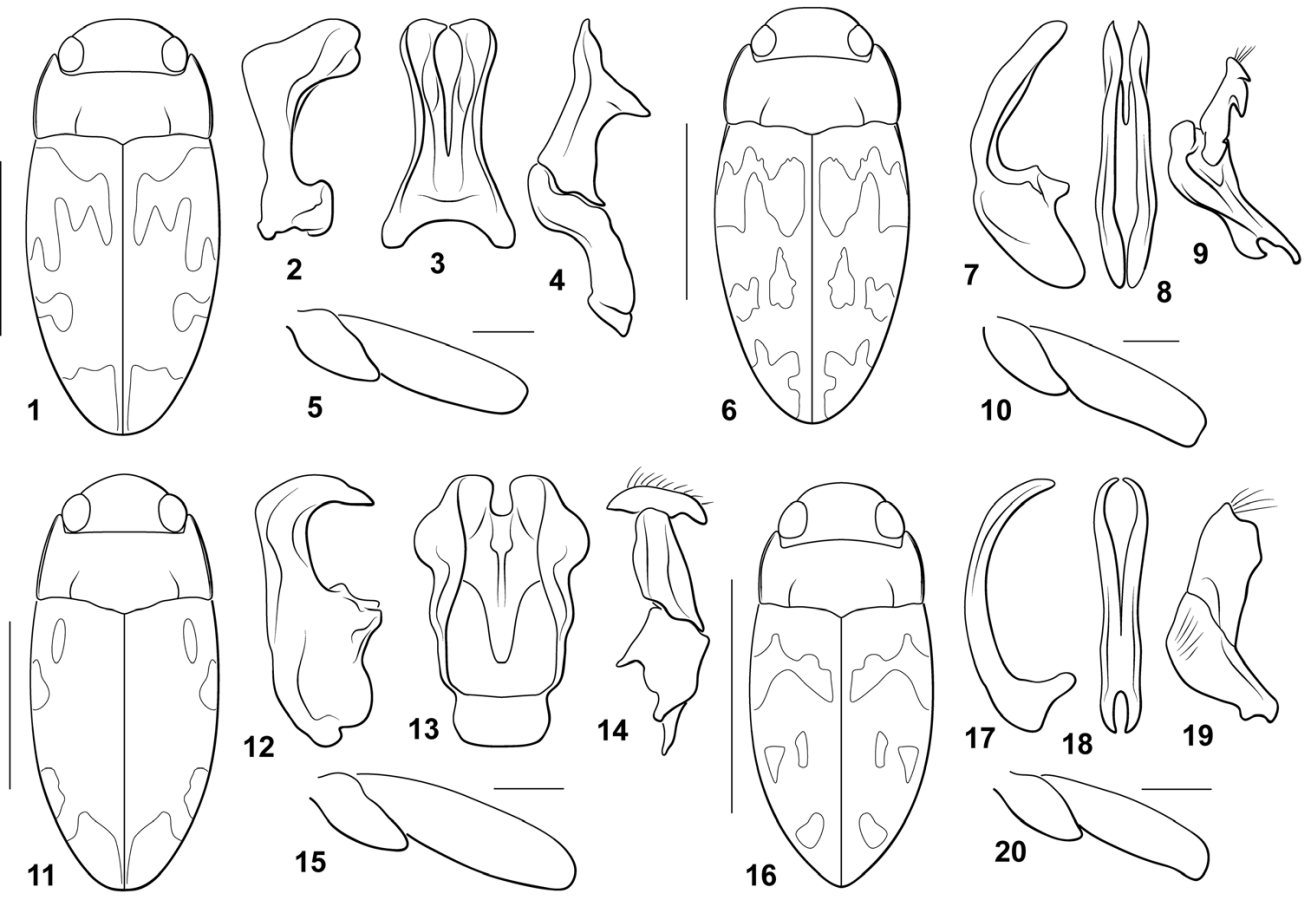
*Bidessodes* species. **1–5** *Bidessodes chlorus* **1** dorsal habitus, scale = 1 mm **2–4** male genitalia **2** median lobe, right lateral aspect **3** median lobe, ventral aspect **4** right lateral lobe, right lateral aspect **5** left metatrochanter and metafemur, anterior aspect, scale = 0.25 mm **6** *Bidessodes erythros* **6** dorsal habitus, scale = 1 mm **7–9** male genitalia **7** median lobe, right lateral aspect **8** median lobe, ventral aspect **9** right lateral lobe, right lateral aspect **10** left metatrochanter and metafemur, anterior aspect, scale = 0.25 mm **11** *Bidessodes leukus* **11** dorsal habitus, scale = 1 mm **12–14** male genitalia **12** median lobe, right lateral aspect **13** median lobe, ventral aspect **14** right lateral lobe, right lateral aspect **15** left metatrochanter and metafemur, anterior aspect, scale = 0.25 mm **16** *Bidessodes melas* **16** dorsal habitus, scale = 1 mm **17** male genitalia **17** median lobe, right lateral aspect **18** median lobe, ventral aspect **19** right lateral lobe, right lateral aspect **20** left metatrochanter and metafemur, anterior aspect, scale = 0.25 mm.

*Bidessodes* are characterized by the following features: (1) a transverse occipital line is absent (e.g. Fig. 1), (2) the anterior clypeal margin is unmodified (Fig. 1), (3) the basal pronotal striae are present (Fig. 1), (4) the basal elytral stria is absent (Fig. 1), (5) the elytral sutural stria is absent (Fig. 1), and (6) the transverse carina across the epipleuron at the humeral angle of the elytron is absent. The genus most similar in general appearance to *Bidessodes* in Bidessini is *Neobidessodes* Hendrich and Balke, 2009, a group of species from Australia previously placed in *Bidessodes*. The main difference between these genera is a series of very fine serrations or denticles along the posterior margins of the abdominal ventrites, present in *Bidessodes* and absent in *Neobidessodes*.

#### Comments.

The genera *Hughbosdineus* and *Youngulus* were proposed by Spangler (1981) and relegated to subgenera of *Bidessodes* by Young (1986). It seems clear, though, that the species were placed in their own genera based on unusual apomorphies rather than clear evidence of phylogenetic isolation. Although there has not been a phylogenetic analysis of the group, these two species appear to be well within the general character-based concept of *Bidessodes*. There is little justification for continued recognition of three subgenera in *Bidessodes*, so, *Hughbosdineus* Spangler, 1981 and *Youngulus* Spangler, 1981 are each placed as junior synonyms of *Bidessodes* Régimbart, 1895 (new synonymies).

#### Key to species of *Bidessodes*

The following key is modified from Young (1986) and Braga and Ferreira-Jr. (2009). Keys to *Bidessodes* have been historically based on male attributes. This key is similarly limited. Females of many species are extremely similar and cannot be easily distinguished without association with males. Much of the key requires dissection of male genitalia, and even with the key the best diagnostic method is to dissect male genitalia and compare with descriptions and images of them. *Bidessodes fragilis* is not keyed given ambiguity about its identity and character combination.

**Table:** 

1	Prosternal process anterior to procoxae distinctly carinate with distinctive setae or spines anteriorly in only males or both males and females	**2**
–	Prosternal process anterior to procoxae not distinctly carinate in either sex, simply rounded or only weakly carinate, without distinctive setae or spines	**3**
2	Prosternal process carinate anterior to procoxae in both males and females; males with distinctive transverse impression across metaventrite behind mesocoxae; metatrochanter and metafemur large, but not conspicuously modified (Fig. 71); length: 2.3–2.6 mm; male median lobe in lateral aspect apically somewhat curved with distinct subapical dorsal expansion (Fig. 68); lateral lobe very broad, apical segment large, broadly subtriangular, oriented obliquely with respect to basal segment (Fig. 70); Bolivia, Brazil, Guyana, and Venezuela (Fig. 104)	***Bidessodes knischi***
–	Prosternal process anterior to procoxae carinate in males but not females; metaventrite not impressed in either sex; metatrochanter large and rounded apically, conspicuously extending beyond ventral margin of metafemur (Fig. 81); length: 2.4–2.9 mm; male median lobe in lateral aspect apically strongly curved without distinct subapical expansion (Fig. 78); lateral lobe with apical segment very large, expanded apically, broadly rounded at apex, linear with respect to small basal segment (Fig. 80); Guyana, Brazil (Fig. 100)	***Bidessodes obscuripennis***
3	Male metatrochanter large, almost square in outline and metafemur enlarged (Fig. 50); male abdominal ventrite VI transversely impressed subapically, apex weakly carinate; male median lobe in lateral aspect with apical portion medially broadly expanded, narrowed to elongate, slender, straight at apex (Fig. 47); length: 2.8–3.1 mm; Colombia, Venezuela, Guyana (Fig. 98)	***Bidessodes franki***
–	Male metatrochanter variable, in some cases slightly modified in males but not conspicuously modified as above in either sex; male abdominal ventrite VI variable; male genitalia different from described above	**4**
4	Male mesotibia distinctly bent at base (as in Fig. 66)	**5**
–	Male mesotibia not bent	**9**
5	Pronotum widest at base, nearly as wide as distance across bases of elytra (Fig. 61); male median lobe with two long branches, each of which is broadly spatulate and trilobed (Fig. 63); lateral lobe with apical segment broad and round (Fig. 64); size small, about 2.2–2.4 mm in length; Brazil and Bolivia (Fig. 103)	Bidessodes jucundus
–	Pronotum widest medially, narrowed posteriorly; male median lobe may be comprised of two long branches, but not apically spatulate and trilobed; lateral lobe with apical segment various, but not broad and round	**6**
6	Last visible abdominal ventrite of male with distinct transverse sulcus; length: 2.6–2.7 mm; Brazil (Fig. 104)	***Bidessodes hamadae***
–	Last visible abdominal ventrite of male unmodified or variously impressed, but without carina or sulcus	**7**
7	Mesofemur of male not expanded; length: 2.2–2.6 mm; Brazil (Fig. 102)	***Bidessodes nessimiani***
–	Mesofemur of male expanded and distinctly swollen in apical 1/3	**8**
8	Elytra dark, without pale fasciae or maculae (Fig. 21); male median lobe in lateral aspect broadly curved, slender with elongate expansion along dorsal margin medially (Fig. 22); lateral lobe short, apical segment broad, apically truncate with broad expansion subapically along dorsal margin (Fig. 24); length: 2.5–2.8 mm; Bolivia and Brazil (Fig. 99)	***Bidessodes acharistus***
–	Elytra with pale fasciae (Fig. 92); male median lobe simple, in lateral aspect shallowly curved, expanded medially along ventral margin (Fig. 93); lateral lobe long, apical segment elongate, sinuate and apically slender and sharply pointed (Fig. 95); length: 2.4–2.8 mm; Brazil (Fig. 103)	***Bidessodes zimmermanni***
9	Abdominal ventrite VI of male deeply impressed on each side, impression medially carinate; pro- and mesotarsomere I of male not laterally expanded, similar to female mesotarsomeres; male median lobe deeply bifid, with each branch with distinctive apical “hooks” in ventral aspect (Fig. 89); size small, length: 2.0 mm (Fig. 87); Brazil and Guyana (Fig. 103)	***Bidessodes s subsignatus***
–	Abdominal ventrite VI of male not or only weakly impressed on each side, usually impressed in an oval or round area subapically or otherwise modified; pro- and mesotarsomere I of male distinctly expanded; male median lobe various, but not deeply bifid with apical “hooks” in ventral aspect; size larger, length > 2.0 mm	**10**
10	Apex of abdominal ventrite VI of male with distinct strigose sculpture or a longitudinal sulcus	**11**
–	Apex of abdominal ventrite VI of male unmodified or variously impressed, but without carina or sulcus	**12**
11	Abdominal ventrite VI of male with an area of coarse strigose sculpture subapically; male median lobe in lateral aspect extremely slender and curved with slender, pointed apical branches (Fig. 42); lateral lobe with apical portion irregularly shaped, subquadrate with apical short lobe (Fig. 43); length: 2.2–2.4 mm; Brazil, Guyana, Suriname, and Venezuela (Fig. 102)	***Bidessodes evanidus***
–	Abdominal ventrite VI of male without sculpturing subapically, sulcate instead; male median lobe with apex in lateral aspect sinuate (Fig. 32); lateral lobe with apical portion oval (Fig. 34); length: 2.4–2.6 mm; Brazil (Fig. 100)	***Bidessodes demarcoi***
12	Male median lobe in lateral aspect with apical portion moderately broad, sublinear, apically slender and very narrowly rounded (Fig. 83); length: 2.5–3.4 mm (Fig. 82); Brazil, French Guiana, and Suriname (Fig. 100)	***Bidessodes semistriatus***
–	Male median lobe not as described above; length < 3.0 mm	**13**
13	Male median lobe robust, curved, apically linear along dorsal margin, broadly expanded along ventral margin (Fig. 56); length: 2.4–2.6 mm; Venezuela, French Guiana (Fig. 100)	***Bidessodes hygrobius***
–	Male median lobe not as described above	**14**
14	Male median lobe in lateral aspect elongate, slender, evenly curved (Fig. 17); size small, length: 1.6–1.7 mm; Venezuela (Fig. 98)	***Bidessodes melas***
–	Male median lobe in lateral aspect robust, or differently shaped; size larger, length > 2.0 mm	**15**
15	Male median lobe in lateral aspect very broad and evenly curved, apically very slender (Fig. 27); length: 2.1–2.6 mm; Venezuela, Guyana, Suriname (Fig. 99)	***Bidessodes charaxinus***
–	Male median lobe in lateral aspect abruptly curved apically or linear, apex robust	**16**
16	Male median lobe in lateral aspect apically straight and broad (Fig. 7); length: 2.3–2.4 mm; Guyana (Fig. 97)	***Bidessodes erythros***
–	Male median lobe in lateral aspect apically abruptly curved	**17**
17	Male median lobe in ventral aspect extremely broad with broad lateral lobes (Fig. 12); length: 2.4 mm; Venezuela (Fig. 97)	***Bidessodes leukus***
–	Male median lobe in ventral aspect not so broad, without broad lateral lobes	**18**
18	Male median lobe in lateral aspect with apex extremely broad and truncate (Fig. 2); length: 2.3–2.5 mm; Suriname (Fig. 97)	***Bidessodes chlorus***
–	Male median lobe in lateral aspect somewhat more slender, apically pointed (Fig. 36); length: 2.6–2.9 mm; Central America (Fig. 101)	***Bidessodes elongatus***

### Bidessodes chlorus sp. n.

Taxon classificationAnimaliaORDOFAMILIA

http://zoobank.org/BD275772-CBAB-47AC-B94F-97470EF62292

Figs 1–5 , 97

#### Type locality.

Suriname, Sipaliwini District, Camp 1, on Kutari River, 2°10.521'N 56°47.244'W.

#### Type material.

Holotype in MIZA, male labeled, “SURINAME Sipaliwini District 2°10.521'N 56°47.244'W: 228m Camp 1, on Kutari River leg.A.E.Z.Short, UV-light 19-24.viii.2010; SR10-0819-LT1 2010 CI-RAP Survey/ SEMC0915810 KUNHM-ENT [barcode label]/ HOLOTYPE *Bidessodes chlorus* Miller, 2016 [red label with black line border].” Paratypes, 6, labeled same as holotype except with different specimen barcode labels and each with “…PARATYPE *Bidessodes chlorus* Miller, 2016 [blue label with black line border].”

#### Diagnosis.

This species does not have a carinate prosternum in either male or female. The prosternal process is longitudinally slightly convex and relatively narrow with the lateral margins distinctly convergent to a pointed apex. The male mesotibia is unmodified. The male metatrochanter and metafemur are unmodified (Fig. 5). The male abdominal ventrite VI is unmodified. The male median lobe in lateral aspect is very small basally with the apical portion abruptly bent and broadly expanded with the apex bilobed (Fig. 2). In ventral aspect the median lobe is very broad basally with the lateral margin concave and the apex broad with a deep, narrow medial emargination (Fig. 3). The basal segment of the lateral lobe is elongate and subsinuate (Fig. 4). The apical segment is elongate and obliquely T-shaped with the apical margin broadly emarginate (Fig. 4). Externally, *Bidessodes chlorus* is similar to many other species of *Bidessodes* with relatively unmodified ventral surfaces and legs in males or females. The main difference between this species and others is the male genitalia. The shape of the male median lobe and lateral lobes as described above (Figs 2–4) are unlike any other species in the genus. The very broad, angled apical section of the median lobe in lateral aspect (Fig. 2) is particularly unique.

#### Description.

*Measurements*. TL = 2.3–2.5 mm, EW = 1.0–1.1 mm, PW = 0.9–1.0 mm, HW = 0.7–0.9 mm, ED = 0.4–0.5 mm, TL/EW = 2.1–2.2, HW/ED = 1.8–1.9. Body shape elongate oval, pronotum widest medially, similar in width to greatest width of elytra, lateral outline discontinuous between pronotum and elytron.

*Coloration* (Fig. 1). Head and pronotum yellow, posterior margin of pronotum medially brown. Elytron with base color brown to dark brown with diffuse, transverse pale regions anteriorly, medially and at apex (Fig. 1); surface not iridescent. Head appendages, legs and ventral surfaces yellow to yellow-orange.

*Sculpture and structure* (Fig. 1). Head with dorsal surface smooth and shiny, impunctate. Pronotum (Fig. 1) smooth and shiny with few micropunctures, broadly distributed; lateral margins broadly rounded, pronotum broadest medially, slightly constricted at posterior angles, about same width as greatest width of elytra; basal striae moderately well impressed, extending about 2/5 distance across pronotum. Elytron (Fig. 1) very broadly curved laterally; shiny, finely microreticulate across surface, irregularly and inconsistently micropunctate. Prosternal process elongate triangular, basally broad and posteriorly convergent to sharply pointed apex; surface weakly convex. Metaventrite process anteriorly produced with weak lateral carinae that do not extend posteriorly; surface of metaventrite smooth and shiny, not carinate or otherwise modified. Metacoxa with lateral portions shiny, not punctate; metacoxal lines elongate, subparallel. Abdominal ventrites smooth, impunctate; ventrite VI smooth, evenly convex, apically pointed.

*Male genitalia*. Median lobe in lateral aspect short and robust, basal portion small, apical portion robust, strongly angulate medially, apically broad and truncate (Fig. 2); median lobe in ventral aspect broad basally, medially constricted and expanded apically with lateral margins each broadly concave, medially deeply and narrowly emarginate, lateral branches apically rounded with small medially-directed point (Fig. 3); lateral lobe in lateral aspect irregular, basal portion elongate, curved and constricted medially along ventral margin, apical portion broad basally, apically broadly and obliquely T-shaped with lateral apices pointed and truncate apex shallowly and irregularly concave (Fig. 4).

*Sexual dimorphism*. Male pro- and mesotarsomeres I-III broader than in female with extensive ventral adhesive setae. Male and female otherwise similar.

*Variation*. Specimens vary in the extent and intensity of coloration on the elytral surface, though all specimens have some degree of maculation.

#### Etymology.

This species is named *chlorus*, Greek for “pale green” after one of the four horsemen of the apocalypse.

#### Distribution.

*Bidessodes chlorus* is known only from southern Suriname (Fig. 97).

#### Habitat.

The type specimens were collected at a UV light. No other natural history information is known about this species.

### Bidessodes erythros sp. n.

Taxon classificationAnimaliaORDOFAMILIA

http://zoobank.org/13FCB617-738B-4272-AA9A-225E7C88B53E

Figs 6–10 , 97

#### Type locality.

Guyana, Region 8, Konawaruk River, Basecamp (blackwater camp), 5°03.884'N 59°12.838'W.

#### Type material.

Holotype in MIZA, male labeled, “GUYANA:Region 8 5°03.884'N 59°12.838'W, 75 m Konawaruk River, Basecamp (blackwater camp): Blackwater crk along margin, with leaf litter leg. Salisbury & La Cruz 10.ix.2014: GY14-0910-02/ SEMC1428326 [barcode label]/ HOLOTYPE *Bidessodes erythros* Miller, 2016 [red label with black line border].” Paratypes, 15, labeled same as holotype except with different specimen barcode labels and each with “…PARATYPE *Bidessodes erythros* Miller, 2016 [blue label with black line border].”

#### Diagnosis.

This species does not have a carinate prosternum in either male or female. The prosternal process is broadly triangular, slightly convex and apically acuminate. The male mesotibia is unmodified. The male metatrochanter and metafemur are unmodified (Fig. 10) and the male last abdominal ventrite is unmodified. The male median lobe in lateral aspect is broadly lobate basally with the apical portion long, sublinear, moderately broad with somewhat undulate dorsal and ventral margins and of somewhat even width to a rounded apex (Fig. 7). In ventral aspect the median lobe is moderately broad and similar in width to the apex which is divided into two long rami separated by a narrow, deep medial emargination, with the apex of each ramus narrowed to a point (Fig. 8). The basal segment of the lateral lobe is elongate and irregularly shaped (Fig. 9). The apical segment is small with the apex characterized by an anteriorly directed spinous process on the dorsal margin (Fig. 9). Externally, *Bidessodes erythros* is similar to many other species of *Bidessodes* with relatively unmodified ventral surfaces and legs in males or females. The main difference between this species and others is the male genitalia as described above. The male median lobe and lateral lobes (Figs 7–9) are unlike any other species in the genus.

#### Description.

*Measurements*. TL = 2.3–2.4 mm, EW = 1.2–1.3 mm, PW = 1.0–1.1 mm, HW = 0.7–0.9 mm, ED = 0.4–0.5 mm, TL/EW = 2.1–2.3, HW/ED = 1.7–1.8. Body shape elongate oval, pronotum widest medially, width less than greatest width of elytra, lateral outline slightly discontinuous between pronotum and elytron, posteriorly somewhat attenuate.

*Coloration* (Fig. 6). Head and pronotum yellow-orange, posterior margin of pronotum medially narrowly brown. Elytron with base color dark brown with weakly differentiated, diffuse, transverse pale fasciae anteriorly, medially and at apex, anterior and medial pale regions meeting in longitudinal areas medially (Fig. 6); surface not iridescent. Head appendages, legs and ventral surfaces yellow to yellow-orange.

*Sculpture and structure* (Fig. 6). Head with dorsal surface smooth and shiny, with extremely fine micropunctures, broadly dispersed. Pronotum (Fig. 6) smooth and shiny with broadly dispersed micropunctures; lateral margins broadly rounded, pronotum broadest medially, slightly constricted at posterior angles, somewhat less in width than greatest width of elytra; basal striae moderately well impressed, extending about 2/5 distance across pronotum. Elytron (Fig. 6) very broadly curved laterally; shiny, finely microreticulate across surface, distinctly punctate. Prosternal process basally broad and posteriorly slightly convergent to broadly pointed apex, lateral margins somewhat straight and convergent; surface approximately flat. Metaventrite process anteriorly produced with weak lateral carinae that do not extend posteriorly; surface of metaventrite smooth and shiny, not carinate or otherwise modified. Metacoxa with lateral portions shiny, not punctate; metacoxal lines elongate, subparallel, apically distinctly convergent; metatrochanter and metafemur relatively unmodified. Abdominal ventrites smooth, impunctate; ventrite VI smooth, evenly convex, apically broadly pointed.

*Male genitalia*. Median lobe in lateral aspect with elongate, oval basal part, apical portion nearly straight, robust, with lateral margins subparallel to rounded apex (Fig. 7); median lobe in ventral aspect broad, lateral margins weakly sinuate, similar in width throughout, apex bifid, each branch apically broadly pointed and slightly curved inward, emargination V-shaped (Fig. 8); lateral lobe in lateral aspect with basal segment elongate, subtriangular with distict apicoventral lobe at base of apical segment; apical segment short and small, apically with truncate and laterally toothed apex, with large, basally-directed tooth on dorsal margin (Fig. 9).

*Sexual dimorphism*. Male pro- and mesotarsomes I-III broader than in female and with extensive ventral adhesive setae. Otherwise males and females similar.

*Variation*. Specimens are relatively consistent in color pattern and other features.

#### Etymology.

This species is named *erythros*, Greek for “red” after one of the four horsemen of the apocalypse.

#### Distribution.

This species is known only from central Guyana (Fig. 97).

#### Habitat.

The type series was collected from a leaf litter area along the margins of a blackwater creek.

### Bidessodes leukus sp. n.

Taxon classificationAnimaliaORDOFAMILIA

http://zoobank.org/D4CA601F-769D-4B0E-B3E1-CE35E70E30E5

Figs 11–15 , 97

#### Type locality.

Venezuela, Amazonas State, Comunidad Caño Gato, Rio Sipapo 4°58.838'N, 67°44.341'W.

#### Type material.

Holotype in MIZA, male labeled, “VENEZUELA: Amazonas State 4°58.838'N, 67°44.341'W: 95m Comunidad Caño Gato Rio Sipapo: 16.i.2009; leg. Short, Miller, Camacho, Joly & Garcia VZ09-0116-01X: along stream/ SM0842868 KUNHM-ENT [barcode label]/ HOLOTYPE *Bidessodes leukus* Miller, 2016 [red label with black line border].”

#### Diagnosis.

This species does not have a carinate prosternum in either male or female. The prosternal process is broad, broadly convex, with the lateral margins convergent to the rounded apex. The male mesotibia is unmodified. The male metatrochanter and metafemur are unmodified (Fig. 15) and the male abdominal ventrite VI is unmodified. The male median lobe in lateral aspect is very broad basally with the apical portion short and abruptly curved (Fig. 12). In ventral aspect the median lobe is very broad with undulate lateral margins and the apex broad and medially emarginate (Fig. 13). The basal segment of the lateral lobe is short and subquadrate with the proximate margin toothed (Fig. 14). The apical segment is elongate and T-shaped with the apical margin broadly curved and with a series of setae (Fig. 14). Externally, *Bidessodes leukus* is similar to many other species of *Bidessodes* with relatively unmodified ventral surfaces and legs in males or females. The main difference between this species and others is the unique male genitalia. The male median lobe and lateral lobes (Figs 12–14) are unlike any other species in the genus. In particular, the median lobe is very broad in ventral aspect (Fig. 12) and robust and strongly curved in lateral aspect (Fig. 13).

#### Description.

*Measurements*. TL = 2.4 mm, EW = 1.1 mm, PW = 1.0 mm, HW = 0.7 mm, ED = 0.4 mm, TL/EW = 2.3, HW/ED = 1.9. Body shape elongate oval, pronotum widest medially, width somewhat less that greatest width of elytra, lateral outline discontinuous between pronotum and elytron, posteriorly somewhat attenuate.

*Coloration* (Fig. 11). Head and pronotum yellow, posterior margin of pronotum medially narrowly brown. Elytron with base color brown with very weakly differentiated, diffuse, transverse pale regions anteriorly, medially and at apex (Fig. 11); surface not iridescent. Head appendages, legs and ventral surfaces yellow to yellow-orange.

*Sculpture and structure* (Fig. 11). Head with dorsal surface smooth and shiny, with extremely fine micropunctures. Pronotum (Fig. 11) smooth and shiny with few broadly dispersed micropunctures; lateral margins broadly rounded, pronotum broadest medially, slightly constricted at posterior angles, about same width as greatest width of elytra; basal striae moderately well impressed, extending to nearly 1/2 distance across pronotum. Elytron (Fig. 11) very broadly curved laterally; shiny, finely microreticulate across surface, impunctate. Prosternal process basally broad and posteriorly slightly convergent to broadly rounded apex, lateral margins broadly rounded; surface approximately flat. Metaventrite process anteriorly produced with weak lateral carinae that do not extend posteriorly; surface of metaventrite smooth and shiny, not carinate or otherwise modified. Metacoxa with lateral portions shiny, not punctate; metacoxal lines elongate, subparallel, apically distinctly convergent. Abdominal ventrites smooth, impunctate; ventrite VI smooth, evenly convex, apically broadly pointed.

*Male genitalia*. Median lobe in lateral aspect with basal portion broad and irregular, apical portion robust, short, abruptly curved at nearly right angle medially, apically narrowed to elongate pointed apex (Fig. 12); median lobe in ventral aspect very broad, apically with lateral margins broadly expanded into lobes laterally, apex bifid (Fig. 13); lateral lobe in lateral aspect irregular, basal portion broad and short, basally with lobes and teeth, apical portion elongate and T-shaped, apex broadly expanded laterally, apical margin curved (Fig. 14).

*Sexual dimorphism*. Females not examined, but male pro- and mesotarsomeres I-III laterally expanded with ventral adhesive setae, similar to other *Bidessodes* species that exhibit this dimorphism.

*Variation*. Only a single male specimen was examined.

#### Etymology.

This species is named *leukus*, Greek for “white” after one of the four horsemen of the apocalypse.

#### Distribution.

*Bidessodes leukus* is known only from southwestern Venezuela (Fig. 97).

#### Habitat.

The type was collected from slow areas along a sandy forest stream with extensive leaf pack.

### Bidessodes melas sp. n.

Taxon classificationAnimaliaORDOFAMILIA

http://zoobank.org/EE844E6C-8E46-4E2B-BCFE-029827906382

Figs 16–20 , 98

#### Type locality.

Venezuela, Amazonas State, Comunidad Caño Gato, Rio Sipapo, 4°58.838'N, 67°44.341'W.

#### Type material.

Holotype in MIZA, male labeled, “VENEZUELA: Amazonas State 4°58.838'N, 67°44.341'W: 95m Comunidad Caño Gato Rio Sipapo: 16.i.2009; leg. Short, Miller, Camacho, Joly & Garcia VZ09-0116-01X: along stream/ SM0842862 KUNHM-ENT [barcode label]/ HOLOTYPE *Bidessodes melas* Miller, 2016 [red label with black line border].” Paratypes, 66, labeled same as holotype except with different specimen barcode labels and each with “…PARATYPE *Bidessodes melas* Miller, 2016 [blue label with black line border].”

#### Diagnosis.

This species does not have a carinate prosternum in either male or female. The prosternal process is longitudinally slightly convex and relatively narrow with the lateral margins distinctly convergent to a pointed apex. The male mesotibia is unmodified. The male metatrochanter and metafemur are unmodified (Fig. 20) and the male last abdominal ventrite is unmodified. The male median lobe in lateral aspect is very small basally with the apical portion long, slender and evenly curved to a narrowly rounded apex (Fig. 17). In ventral aspect the median lobe is slender basally with the lateral margins expanded apically, divided into two long, slender curved rami that bend medially apically (Fig. 18). The basal segment of the lateral lobe is broadly obliquely triangular (Fig. 19). The apical segment is broad and obliquely subrectangular with the apex obliquely subtruncate (Fig. 19). Externally, *Bidessodes melas* is similar to many other species of *Bidessodes* with relatively unmodified ventral surfaces and legs in males or females. The main difference between this species and others is the male genitalia. The slender, elongate, longitudinally emarginate male median lobe and broad and uniquely shaped lateral lobes (Figs 17–19) are unlike any other species in the genus. This is also one of the smallest species in the genus.

#### Description.

*Measurements*. TL = 1.6–1.7 mm, EW = 0.8–0.9 mm, PW = 0.7–0.8 mm, HW =0.5–0.6 mm, ED = 0.3–0.4 mm, TL/EW = 2.1–2.3, HW/ED = 1.7–1.8. Body shape elongate oval, pronotum widest medially, width somewhat less than greatest width of elytra, lateral outline discontinuous between pronotum and elytron.

*Coloration* (Fig. 16). Head and pronotum yellow, posterior margin of pronotum medially narrowly brown. Elytron with base color brown with diffuse, transverse pale regions anteriorly, medially and at apex (Fig. 16); surface slightly but distinctly purplish iridescent. Head appendages, legs and ventral surfaces yellow to yellow-orange.

*Sculpture and structure* (Fig. 16). Head with dorsal surface smooth and shiny, with extremely fine micropunctures. Pronotum (Fig. 16) smooth and shiny with few broadly dispersed micropunctures; lateral margins broadly rounded, pronotum broadest medially, slightly constricted at posterior angles, about same width as greatest width of elytra; basal striae moderately well impressed, extending to nearly 1/2 distance across pronotum. Elytron (Fig. 16) very broadly curved laterally; shiny, finely microreticulate across surface, impunctate. Prosternal process elongate, basally broad and posteriorly convergent to narrowly rounded apex, lateral margins broadly rounded; surface approximately flat. Metaventrite process anteriorly produced with weak lateral carinae that do not extend posteriorly; surface of metaventrite smooth and shiny, not carinate or otherwise modified. Metacoxa with lateral portions shiny, not punctate; metacoxal lines elongate, subparallel. Abdominal ventrites smooth, impunctate; ventrite VI smooth, evenly convex, apically pointed.

*Male genitalia*. Median lobe in lateral aspect with small basal portion, apical portion elongate, slender, broadly and evenly curved to narrowly rounded apex (Fig. 17); median lobe in ventral aspect deeply bifid into two elongate, slender rami, apically pointed and with apices curved towards each other apically (Fig. 18); lateral lobe in lateral aspect very robust, basal and apical portions similar in length and width, apical portion broad, apically truncate and toothed (Fig. 19).

*Sexual dimorphism*. Male pro- and mesotarsomeres I-III broader than in female with extensive ventral adhesive setae. Male and female otherwise similar.

*Variation*. Specimens vary in the intensity and extent of elytral maculae which are not strongly evident in any specimens.

#### Etymology.

This species is named *melas*, Greek for “black” after one of the four horsemen of the apocalypse.

#### Distribution.

This species is known only from southwestern Venezuela (Fig. 98).

#### Habitat.

The type series was collected from leaf pack in slow areas along a sandy forest stream.

### Bidessodes acharistus

Taxon classificationAnimaliaORDOFAMILIA

Young, 1986

Figs 21–25 , 99

Bidessodes (Bidessodes) acharistus Young, 1986:217; Biström, 1988:7; Nilsson, 2016:98.

#### Diagnosis.

**Figures 21–40.**
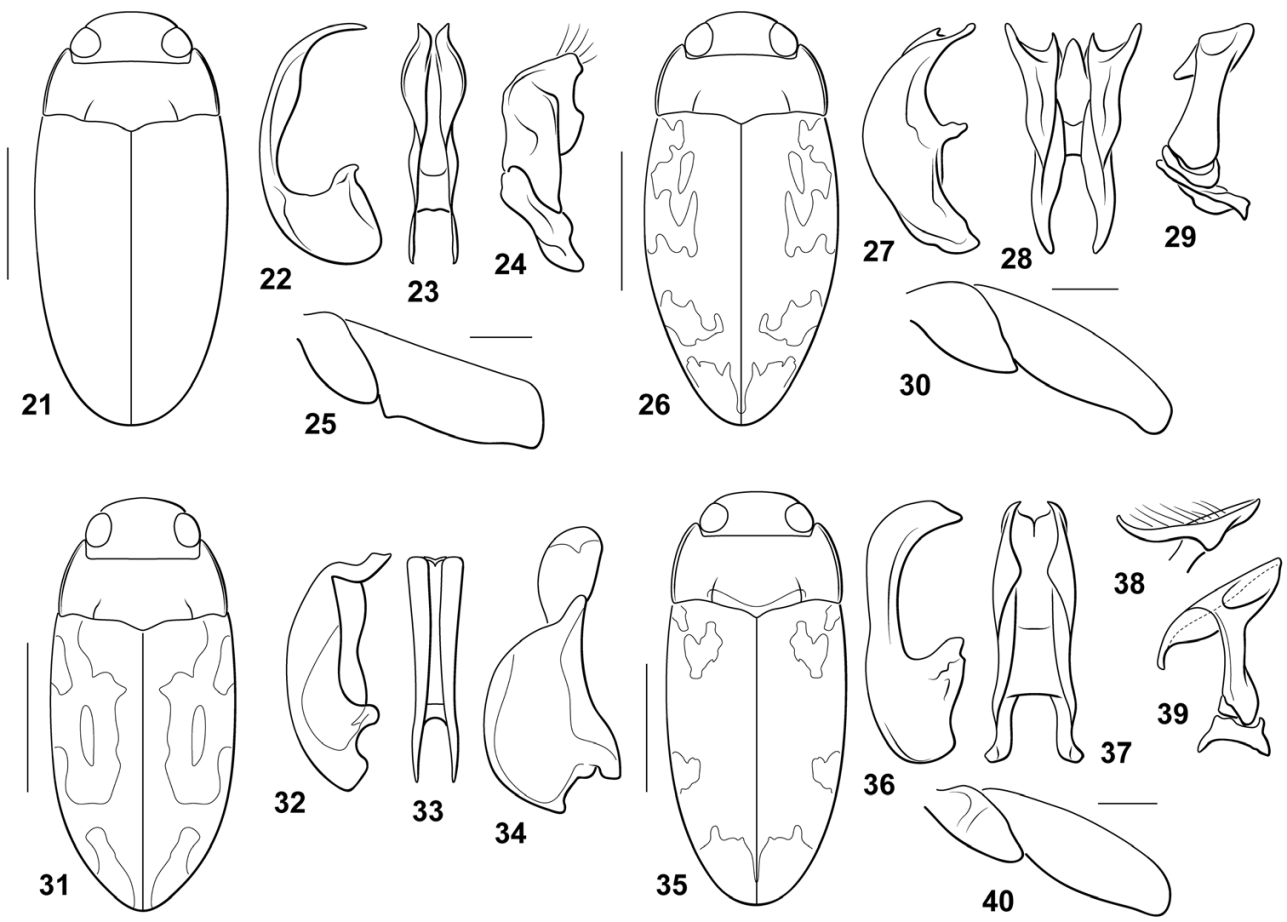
*Bidessodes* species. **21–25** *Bidessodes acharistus* **21** dorsal habitus, scale = 1 mm **22** male genitalia **22** median lobe, right lateral aspect **23** median lobe, ventral aspect **24** right lateral lobe, right lateral aspect **25** left metatrochanter and metafemur, anterior aspect, scale = 0.25 mm **26–30** *Bidessodes charaxinus* **26** dorsal habitus, scale = 1 mm **27** male genitalia **27** median lobe, right lateral aspect **28** median lobe, ventral aspect **29** right lateral lobe, right lateral aspect **30** left metatrochanter and metafemur, anterior aspect, scale = 0.25 mm **31–34** *Bidessodes demarcoi* **31** dorsal habitus, scale = 1 mm **32** male genitalia **32** median lobe, right lateral aspect **33** median lobe, ventral aspect **34** right lateral lobe, right lateral aspect **35–40** *Bidessodes elongatus* **35** dorsal habitus, scale = 1 mm **36** male genitalia **36** median lobe, right lateral aspect **37** median lobe, ventral aspect **38** apex of right lateral lobe, apical aspect **39** right lateral lobe, right lateral aspect **40** left metatrochanter and metafemur, anterior aspect, scale = 0.25 mm.

This species does not have a carinate prosternum in either male or female. The prosternal process is flat and parallel-sided with the apex broadly acuminate. The male mesotibia is basally bent. The male metatrochanter and metafemur are broad, the metafemur is apically truncate with a distinct denticle along the ventral margin near the apex of the metatrochanter (Fig. 25). The male abdominal ventrite VI is apically slightly impressed medially. The male median lobe in lateral aspect is moderately broad basally with an elongate, broadly curved, slender apical portion that is medially somewhat expanded (Fig. 22). In ventral aspect the median lobe is slender and deeply bifid with each ramus sinuate and subapically expanded and ending in a single pointed process (Fig. 23). The basal segment of the lateral lobe is short and oblique (Fig. 24). The apical segment is very broad and has a broad dorsal expansion apically (Fig. 24). Specimens are relatively immaculate (Fig. 21).

#### Distribution.

Known from few localities in Brazil and Bolivia (Fig. 99).

### Bidessodes charaxinus

Taxon classificationAnimaliaORDOFAMILIA

Young, 1986

Figs 26–30 , 99

Bidessodes (Bidessodes) charaxinus Young, 1986: 213; Biström, 1988: 7; Nilsson, 2016: 98.

#### Diagnosis.

This species does not have a carinate prosternum in either male or female. The prosternal process is impressed longitudinally. The lateral margins are slightly convergent apically to the broadly pointed apex. The male mesotibia is elongate, curved, not basally bent. The male metatrochanter and metafemur are not noticeably modified (Fig. 30). The male abdominal ventrite VI is apically somewhat impressed. The male median lobe in lateral aspect is broad with a broad elongate basal region and an apical region that is broad basally and abruptly tapered to an elongate slender apex with a distinct subapical pointed process representing the apicomedial portion of each ramus (Fig. 27). In ventral aspect the median lobe is complex and deeply bifid with each ramus elongate, broad and apically terminating in two sharply pointed processes, and also with a medial terminal lobe between each lateral ramus (Fig. 28). The basal segment of the lateral lobe is small, irregular, and transverse (Fig. 29). The apical segment is broad and complex, terminating in a broad, transverse structure (Fig. 29). Specimens are relatively large and maculate (Fig. 26).

#### Distribution.

Known from northern South America (Venezuela, Suriname, Guyana, French Guiana) south into Brazil (Fig. 99).

### Bidessodes demarcoi

Taxon classificationAnimaliaORDOFAMILIA

Braga and Ferreira-Jr., 2009

Figs 31–34 , 100

Bidessodes (Bidessodes) demarcoi Braga and Ferreira-Jr., 2009: 46; Nilsson, 2016: 98.

#### Diagnosis.

*Bidessodes demarcoi* does not have a carinate prosternum in males or females. The prosternal process is relatively flat, the lateral margins are slightly convergent to the broadly pointed apex. The male mesotibia is unmodified. The male metatrochanter and metafemur are not modified. The male abdominal ventrite VI is sulcate apically. The male median lobe in lateral aspect is broad and broadly curved throughout its length to a sinuate, apically truncate apical region (Fig. 32). In ventral aspect the median lobe is moderately broad with each lateral margin straight, apically slightly divergent and rounded with medial shallow emargination (Fig. 33). The lateral lobe in lateral aspect is extremely broad with a broadly rounded basal portion and the apical portion smaller, but broadly rounded (Fig. 34). Specimens are maculate on the elytra (Fig. 31).

#### Distribution.

Known from north-central Brazil (Fig. 100).

### Bidessodes elongatus

Taxon classificationAnimaliaORDOFAMILIA

(Sharp, 1882)

Figs 35–40 , 101

Bidessus elongatus Sharp, 1882:25; Blackwelder, 1944;76.
Bidessus (Bidessodes) elongatus , Zimmermann, 1919: 61; 1921: 200.
Bidessodes elongatus , Young: 1969: 2.
Bidessodes (Bidessodes) elongatus , Young, 1986: 216; Biström, 1988: 7; Nilsson, 2016: 98.

#### Diagnosis.

This species does not have a carinate prosternum in either male or female. The prosternal process is somewhat medially longitudinally sulcate, the lateral margins are slightly convergent to the rounded apex. The male mesotibia is unmodified. The male metatrochanter is medially transversely somewhat ridged, but the metafemur is not noticeably modified (Fig. 40). The male abdominal ventrite VI is apically and laterally slightly impressed. The male median lobe is basally moderately broad and apically robust and strongly curved to a narrowly rounded apex (Fig. 36). In ventral aspect it is broad and laterally broadly sinuate with the apex shallowly emarginate with each ramus short and sharply pointed (Fig. 37). The basal segment of the lateral lobe is small and transverse (Fig. 38). The apical segment is formed as a slender stalk basally with a very large, transverse apical lobe that is abruptly curved on the ventral apex (Fig. 39). Specimens are elongate slender with maculate elytra (Fig. 35).

#### Discussion.

Sharp (1882) mentioned that this species, “… will no doubt form a distinct genus.” That eventually proved to be the case as *Bidessus elongatus* became the type of *Bidessodes*.

#### Distribution.

The type locality for this species is somewhat ambiguous. The specimens were evidently collected by Champion, but the localities, “Paso Antonio” and “Tortola” are not easily identified today. In a letter from Champion (http://james-champion.com/diary-2012/thursday-14th-june-2012-letter-13th-march-1881/) he mentions the “Rio Michotoya,” which is more easily located, and the star on the map included here reflects that locality (Fig. 101). Known from Guatemala and Costa Rica (Fig. 101).

### Bidessodes evanidus

Taxon classificationAnimaliaORDOFAMILIA

Young, 1986

Figs 41–45 , 102

Bidessodes (Bidessodes) evanidus Young, 1986: 212; Biström, 1988: 7; Nilsson, 2016: 98.

#### Diagnosis.

**Figures 41–60.**
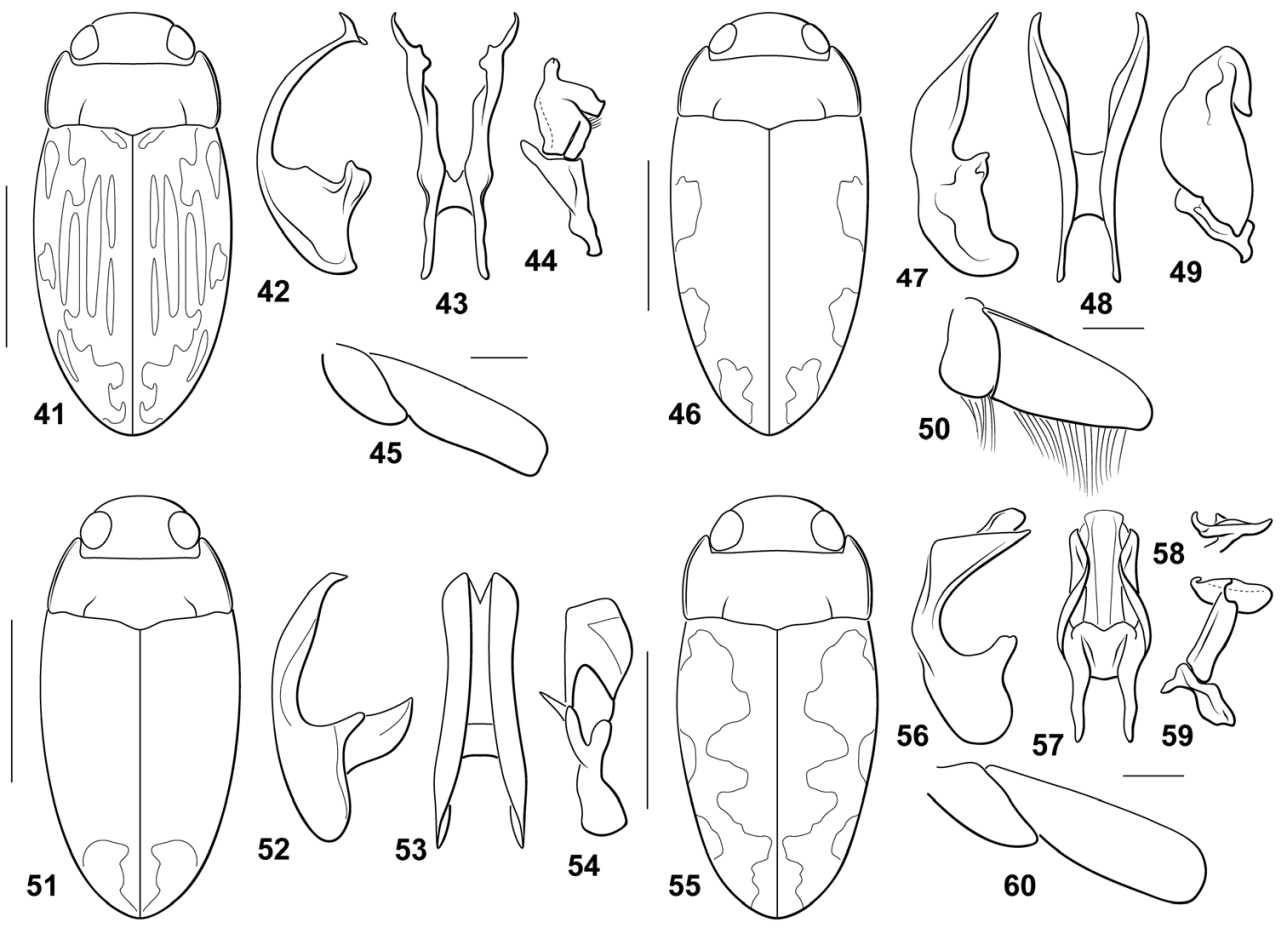
*Bidessodes* species. **41–45** *Bidessodes evanidus* **41** dorsal habitus, scale = 1 mm **42** male genitalia **42** median lobe, right lateral aspect **43** median lobe, ventral aspect **44** right lateral lobe, right lateral aspect **45** left metatrochanter and metafemur, anterior aspect, scale = 0.25 mm **46–50** *Bidessodes franki* **46** dorsal habitus, scale = 1 mm **47** male genitalia **47** median lobe, right lateral 6spect **48** median lobe, ventral aspect **49** right lateral lobe, right lateral aspect **50** left metatrochanter and metafemur, anterior aspect, scale = 0.25 mm **51–54** *Bidessodes hamadae* **51** dorsal habitus, scale = 1 mm **52** male genitalia **52** median lobe, right lateral aspect **53** median lobe, ventral aspect **54** right lateral lobe, right lateral aspect **55–60** *Bidessodes hygrobius* **55** dorsal habitus, scale = 1 mm **56** male genitalia **56** median lobe, right lateral aspect **57** median lobe, ventral aspect **58** right lateral lobe apex, apical aspect **59** right lateral lobe, right lateral aspect **60** left metatrochanter and metafemur, anterior aspect, scale = 0.25 mm.

This species does not have a carinate prosternum in either male or female. The prosternal process is flat, the lateral margins are slightly convergent to the apically rounded apex. The male mesotibia is unmodified. The male metafemur and metatrochanter are not noticeably modified (Fig. 45). The male abdominal ventrite VI is apically and laterally somewhat impressed. The male median lobe is basally broad, but apically very slender with an apical broad, denticulate expansion (Fig. 42). In ventral aspect it is deeply bifid with each branch slender and apically irregular, and sharply angulate (Fig. 43). The basal segment of the lateral lobe is moderately small and elongate triangular. The apical segment is broad and extremely irregular with a prominent apical, finger-like lobe (Fig. 44). Specimens are elongate oval with the elytra longitudinally vittate (Fig. 41);

#### Distribution.

Known from lowland South America from northern Venezuela to southeastern Brazil (Fig. 102).

### Bidessodes fragilis

Taxon classificationAnimaliaORDOFAMILIA

Régimbart, 1900

Fig. 103

Bidessodes fragilis Régimbart, 1900: 530; Blackwelder, 1944: 76; Young, 1969: 2.
Bidessus (Bidessodes) fragilis , Zimmermann, 1919:61;1921:200.
Bidessodes (Bidessodes) fragilis , Young, 1986: 219; Biström, 1988: 7; Nilsson, 2016: 98.

#### Diagnosis.

According to the original description and Young (1986), this species has the clypeus thickened but not margined, the pronotum has punctation similar to the head, the pronotal plicae extend about 1/3 distance across pronotum, the apex of the prosternal process is truncate, not acuminate, the prosternum and prosternal process are not carinate or spinous, and the last abdominal ventrite is not modified.

#### Discussion.

The holotype is a female (Young 1986). Young (1986) was unable to identify this species, but thought it close to *Bidessodes semistriatus* and *Bidessodes knischi*, but also thought *Bidessodes acharistus* may represent the species.

#### Distribution.

Only known from the type locality in Paraguay (Fig. 103).

### Bidessodes franki

Taxon classificationAnimaliaORDOFAMILIA

(Spangler, 1981)

Figs 46–50 , 98

Youngulus franki Spangler, 1981:71.
Bidessodes (Youngulus) franki , Young, 1986: 209; Biström, 1988: 7; Nilsson, 2016: 98.

#### Diagnosis.

Males and females do not have a medially modified prosternum. The prosternal process is flat, the lateral margins are convergent to the broadly pointed apex. The male mesotibia is unmodified. *Bidessodes franki* are unique in having males with an extremely broad metatrochanter and metafemur with a distinctive fringe of setae along their posterior margins (Fig. 50). The male abdominal ventrite VI is apically distinctly impressed, and broadly impressed laterally. The male genitalia are distinctive with the median lobe in ventral aspect deeply bifid with each branch unforked and tapered to a point (Fig. 47). In lateral aspect the median lobe is medially very broad and apically strongly tapered and straight to a sharp apex (Fig. 48). The lateral lobe has a reduced basal segment and the apical segment large and irregularly margined with a distinctive lobe on the apicodorsal margin that is directed basally (Fig. 49). Specimens are robust, relatively large and have maculate elytra (Fig. 46).

#### Distribution.

Found across northern South America from central Colombia to southern Suriname (Fig. 98).

### Bidessodes hamadae

Taxon classificationAnimaliaORDOFAMILIA

Braga and Ferreira-Jr., 2009

Figs 51–54 , 104

Bidessodes (Bidessodes) hamadae Braga and Ferreira-Jr., 2009: 46; Nilsson, 2016: 98.

#### Diagnosis.

Specimens do not have modified prosternum. The prosternal process is flat with the lateral margins somewhat convex and the apex acuminate. The male mesotibia is bent basally. The metafemur and metatrochanter are unmodified. The male abdominal ventrite VI is impressed and sulcate apically. The male median lobe in lateral aspect is basally elongate with an elongate dorsally-directed flange, the apical portion is elongate, medially slightly expanded but nearly straight with the apex slightly hooked dorsally (Fig. 52). In ventral aspect the median lobe is broad with the lateral rami moderately broad, divergent basally, apically obliquely truncate with medial shallow emargination between the apices (Fig. 53). The lateral lobe has the apical segment irregular, broad and apically truncate. The basal portion is elongate Y-shaped (Fig. 54). Specimens are elongate-slender and relatively immaculate with an indistinct apical pale spot (Fig. 51).

#### Discussion.

Only females were examined for this study and the figures of the male genitalia (Figs 52–54) are redrawn from Braga and Ferreira-Jr. (2009).

#### Distribution.

Known from north-central Brazil (Fig. 104).

### Bidessodes hygrobius

Taxon classificationAnimaliaORDOFAMILIA

Young, 1986

Figs 55–60 , 100

Bidessodes (Bidessodes) hygrobius Young, 1986: 216; Biström, 1988: 7; Nilsson, 2016: 98.

#### Diagnosis.

This species does not have a carinate prosternum in either male or female. The prosternal process is longitudinally approximately flat, the lateral margins are subparallel, the apex is pointed. The male mesotibia and the male metafemur and metatrochanter are not noticeably modified (Fig. 60). The male abdominal ventrite VI is unmodified. The male median lobe is robust in lateral aspect, abruptly curved, apically pointed with a distinct ventral expanded angulation and a subapical projecting lobe with the apex elongate pointed (Fig. 56). In ventral aspect it is similarly robust with sinuate lateral margins (Fig. 57). The basal segment of the lateral lobe is small and irregular (Fig. 58). The apical segment is extremely irregular with a prominent transverse apical lobe which is hooked on the ventral apex (Fig. 59). Specimens are robust with maculate elytra (Fig. 55).

#### Distribution.

Known from lowland Venezuela and French Guiana (Fig. 100).

### Bidessodes jucundus

Taxon classificationAnimaliaORDOFAMILIA

Young, 1986

Figs 61–66 , 103

Bidessodes (Bidessodes) jucundus Young, 1986: 209; Biström, 1988: 7; Nilsson, 2016: 98.

#### Diagnosis.

**Figures 61–81.**
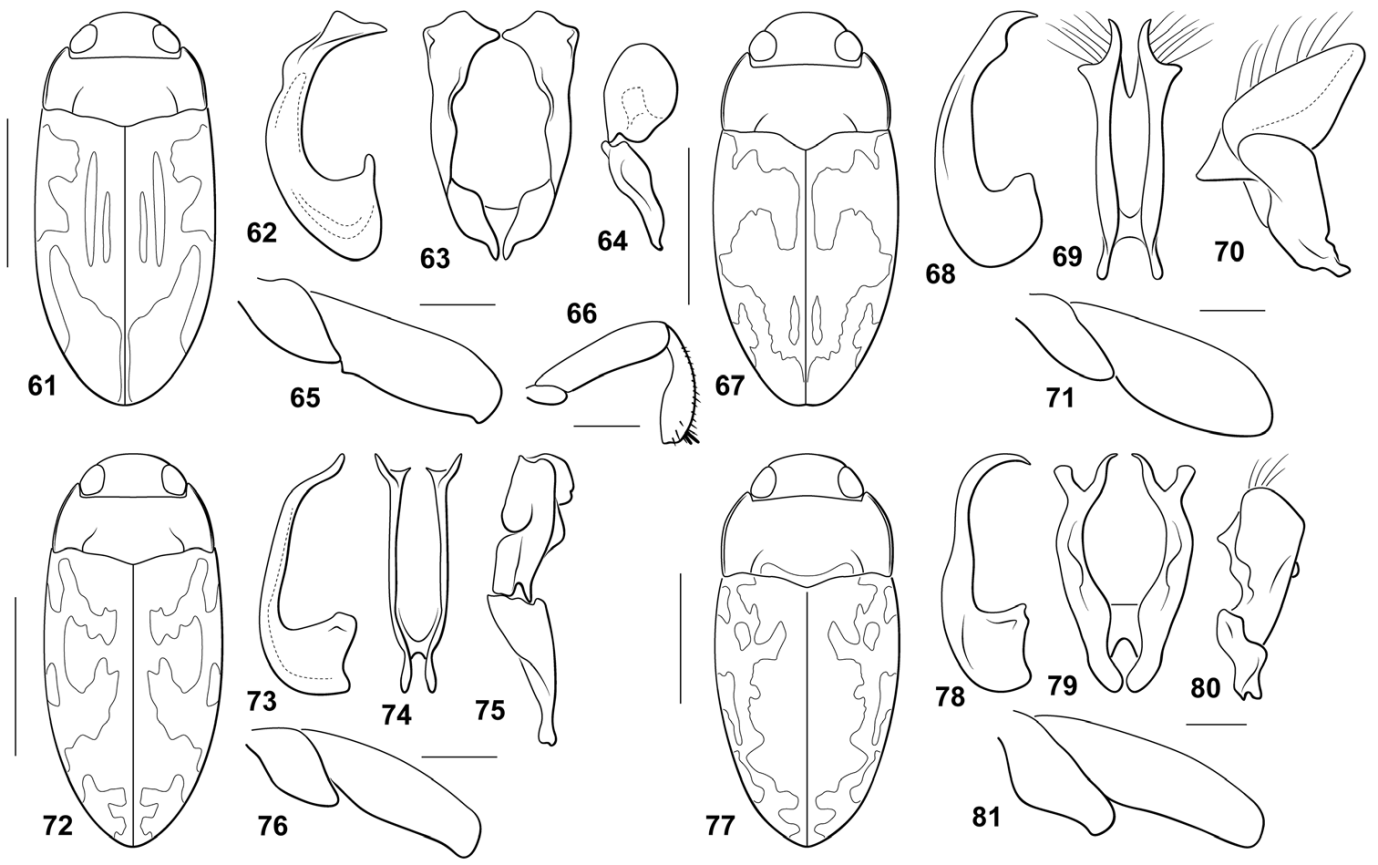
*Bidessodes* species. **61–66** *Bidessodes jucundus* **61** dorsal habitus, scale = 1 mm **62** male genitalia **62** median lobe, right lateral aspect **63** median lobe, ventral aspect **64** right lateral lobe, right lateral aspect **65** left metatrochanter and metafemur, anterior aspect, scale = 0.25 mm **66** left mesotrochanter, mesofemur and mesotibia, anterior aspect, scale = 0.25 mm **67–71** *Bidessodes knischi* **67** dorsal habitus, scale = 1 mm **68** male genitalia **68** median lobe, right lateral aspect **69** median lobe, ventral aspect **70** right lateral lobe, right lateral aspect **71** left metatrochanter and metafemur, anterior aspect, scale = 0.25 mm **72–76** *Bidessodes nessimiani* **72** dorsal habitus, scale = 1 mm **73** male genitalia **73** median lobe, right lateral aspect **74** median lobe, ventral aspect **75** right lateral lobe, right lateral aspect **76** left metatrochanter and metafemur, anterior aspect, scale = 0.25 mm **77–81** *Bidessodes obscuripennis* **77** dorsal habitus, scale = 1 mm **78** male genitalia **78** median lobe, right lateral aspect **79** median lobe, ventral aspect **80** right lateral lobe, right lateral aspect **81** left metatrochanter and metafemur, anterior aspect, scale = 0.25 mm.

This species does not have a carinate prosternum in either male or female. The prosternal process is longitudinally slightly impressed, the lateral margins are convergent to the pointed apex. The male mesotibia is basally abruptly curved (Fig. 66). The male metafemur is moderately broad and has a distinct denticle along the ventral margin apically and another near the apex of the trochanter (Fig. 65). Unlike other species, the lateral pronotal margins are not strongly curved (Fig. 61). The male abdominal ventrite VI is unmodified. The male median lobe is deeply bifurcate with each branch narrow and apically broadly expanded and spatulate in ventral aspect (Fig. 63). In lateral aspect the median lobe is narrow and abruptly and evenly curved (Fig. 62). The basal segment of the lateral lobe is very slender and small, and the apical segment is large and broadly round (Fig. 64).

#### Distribution.

The species is known from Brazil and Bolivia (Fig. 103). Young (1986) mentioned a potential specimen from Panama, but this seems unlikely.

### Bidessodes knischi

Taxon classificationAnimaliaORDOFAMILIA

(Zimmermann, 1921)

Figs 67–71 , 104

Bidessus (Bidessodes) knischi Zimmermann, 1921: 198; Blackwelder, 1944: 76.
Bidessodes knischi , Young, 1969: 2; 1986: 209.
Hughbosdinius leechi Spangler, 1981: 67; synonymy by Young 1986: 206.
Bidessodes (Hughbosdinius) knischi , Young, 1986: 209; Biström, 1988: 7; Nilsson, 2016: 98.

#### Diagnosis.

This species differs from all other species in having the base of the prosternal process distinctly carinate to tectiform in both sexes. In males the ridge anteriorly is flattened and distinctly setose with those setae anteriorly distinctly forked. This species and *Bidessodes obscuripennis* each have the prosternal process basally carinate, at least in males, but they are otherwise rather different. In *Bidessodes obscuripennis*, only males are carinate. The prosternal process is flat and broad, the lateral margins are slightly convergent to the broadly rounded apex. Males of *Bidessodes knischi* have the metaventrite with a prominent transverse groove. The male mesotibia is unmodified. The male metafemur is very broad and rounded (Fig. 71). The male abdominal ventrite VI is distinctly impressed apically. The male genitalia are distinctive. The median lobe in ventral aspect is apically bifid with each branch obliquely bifurcated (Fig. 69). In lateral aspect the median lobe is broadly curved and apically sharply pointed with a distinct expansion along the ventral margin corresponding with the lateral branch of each bifurcation (Fig. 68). The lateral lobe is extremely broad with the apical segment very large and broadly triangular (Fig. 70). Specimens are large and robust with maculate elytra (Fig. 67).

#### Distribution.

The types were collected from Mato Grosso, Brazil with other specimens collected from lowland areas of South America from Venezuela and Guyana south to Bolivia (Fig. 104).

### Bidessodes nessimiani

Taxon classificationAnimaliaORDOFAMILIA

Braga and Ferreira-Jr., 2009

Figs 72–76 , 102

Bidessodes (Bidessodes) nessimiani Braga and Ferreira-Jr., 2009: 44; Nilsson 2016: 98.

#### Diagnosis.

This species lacks modifications to the prosternum and male metathoracic legs. The prosternal process is approximately parallel sided with the apex rounded. The male mesotibia is bent basally. The male metafemur and metatrochanter are unmodified (Fig. 76). The male abdominal ventrite VI is unmodified. The male median lobe in lateral aspect is basally small and subtriangular with the apical portion slender, sublinear, medially and apically recurved (Fig. 73). In ventral aspect the median lobe is broad with the lateral rami extremely slender, apically slender and pointed obliquely with medial broad emargination (Fig. 74). The lateral lobe in lateral aspect is long and complex. The apical portion is broad, apically truncate and variously and broadly lobed on dorsal and ventral margins. The basal portion is slender and elongate sub-triangular (Fig. 75). Specimens are broad with maculate elytra (Fig. 72).

#### Distribution.

Known from north-central Brazil (Fig. 102).

### Bidessodes obscuripennis

Taxon classificationAnimaliaORDOFAMILIA

(Zimmermann, 1921)

Figs 77–81 , 100

Bidessus (Bidessodes) obscuripennis Zimmermann, 1921: 19; Blackwelder 1944: 76.
Bidessodes obscuripennis , Young 1969: 2.
Bidessodes (Hughbosdinius) obscuripennis , Young 1986: 209; Biström 1988: 7; Nilsson 2016: 98.

#### Diagnosis.

The species differs from others in having the prosternal process anteriorly carinate with distinctive setae in males. Females have the prosternal process unmodified. This species and *Bidessodes knischi* each have the prosternal process basally carinate, at least in males, but they are otherwise rather different. The prosternal process is narrow, flat, with the lateral margins slightly convergent to the pointed apex. The metaventrite is not transversely grooved. The male mesotibia is unmodified. The metatrochanter in males is exceptionally large, distinctly offset and prominent apically (Fig. 81). The male abdominal ventrite VI is apically broadly impressed. The male genitalia are also distinctive. In lateral aspect the median lobe is slender and apically abruptly curved (Fig. 78). In ventral aspect the median lobe is deeply bifid, each branch ending in a bifurcation. The medial branch of each bifurcation is sinuate and apically pointed, the lateral branch is broad and apically subtruncate (Fig. 79). The lateral lobe has the apical segment extremely broad and irregularly margined (Fig. 80). Specimens are robust with complex maculae on the elytra (Fig. 77).

#### Distribution.

Known from Guyana and western Brazil (Fig. 100).

### Bidessodes semistriatus

Taxon classificationAnimaliaORDOFAMILIA

Régimbart, 1900

Figs 82–86 , 100

Bidessodes semistriatus Régimbart, 1900: 529; Blackwelder 1944: 76.
Bidessus (Bidessodes) semistriatus , Zimmermann, 1919: 61;1921: 200.
Bidessodes (Bidessodes) semistriatus , Young 1986: 213; Biström, 1988: 7; Nilsson, 2016: 98.

#### Diagnosis.

**Figures 82–96.**
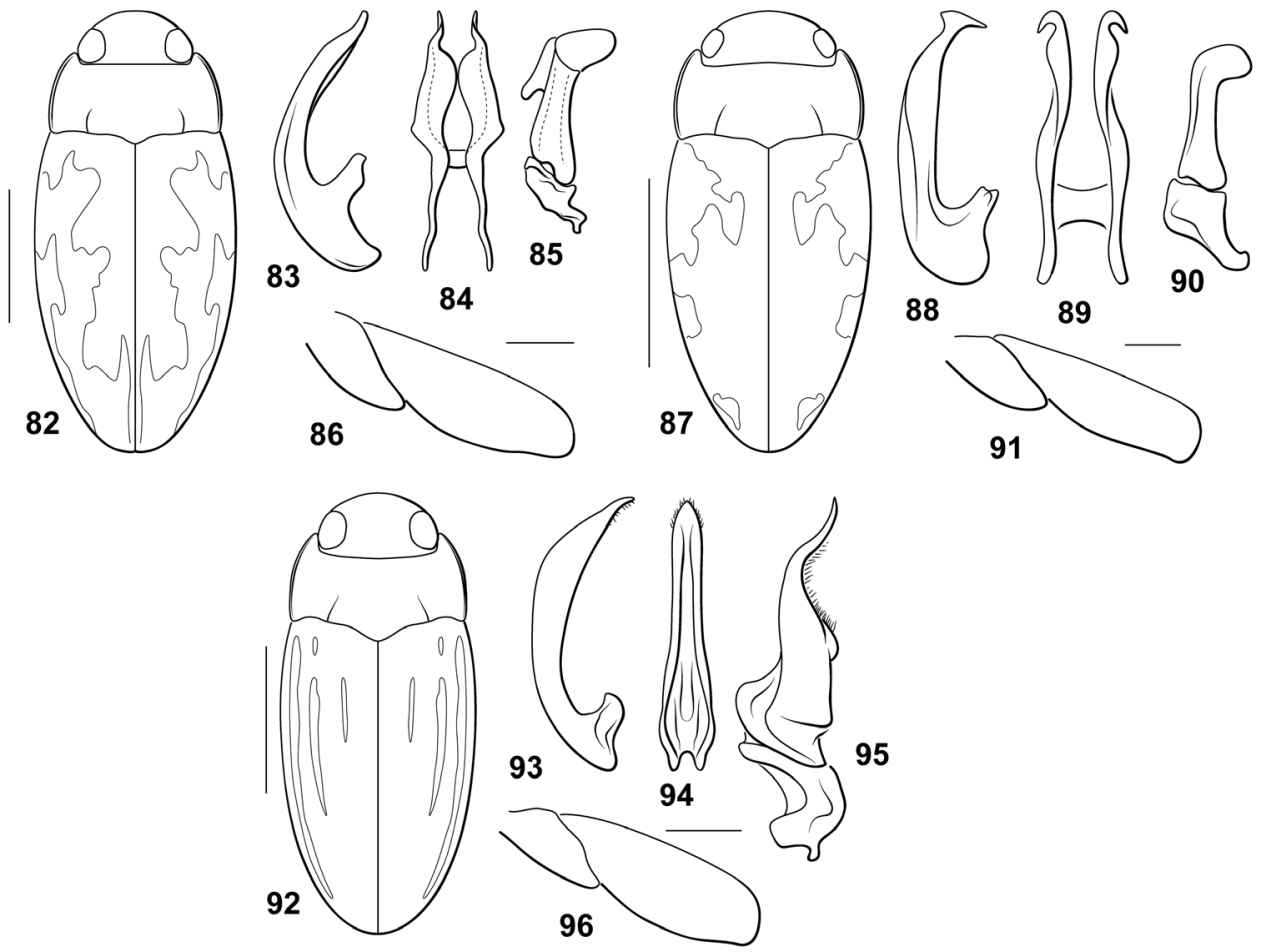
*Bidessodes* species. **82–86** *Bidessodes semistriatus* **82** dorsal habitus, scale = 1 mm **83** male genitalia **83** median lobe, right lateral aspect **84** median lobe, ventral aspect **85** right lateral lobe, right lateral aspect **86** left metatrochanter and metafemur, anterior aspect, scale = 0.25 mm **87–91** *Bidessodes subsignatus* **87** dorsal habitus, scale = 1 mm **88** male genitalia **88** median lobe, right lateral aspect **89** median lobe, ventral aspect **90** right lateral lobe, right lateral aspect **91** left metatrochanter and metafemur, anterior aspect, scale = 0.25 mm **92–96** *Bidessodes zimmermanni* **92** dorsal habitus, scale = 1 mm **93** male genitalia **93** median lobe, right lateral aspect **94** median lobe, ventral aspect **95** right lateral lobe, right lateral aspect **96** left metatrochanter and metafemur, anterior aspect, scale = 0.25 mm.

This species lacks a carinate and spinous prosternum in males and females. The prosternal process is narrow, flat, with the lateral margins subparallel with the apex rounded. The male mesotibia, metafemur and metatrochanter are not modified (Fig. 86). The male abdominal ventrite VI is apically impressed. The male median lobe in lateral aspect is evenly curved (Fig. 83). In ventral aspect the median lobe is deeply bifid and broad, with the lateral rami broad and apically slender, sinuate and apically pointed (Fig. 84). The lateral lobe in lateral aspect is apically broadly T-shaped with the dorsal portion of the “T” broadly lobate (Fig. 85). Specimens are elongate-slender with maculate elytra (Fig. 82).

#### Distribution.

Known from Guyana, Suriname and Brazil (Fig. 100).

### Bidessodes subsignatus

Taxon classificationAnimaliaORDOFAMILIA

(Zimmermann, 1921)

Figs 87–91 , 103

Bidessus (Bidessodes) subsignatus Zimmermann, 1921: 199; Blackwelder, 1944: 76.
Bidessodes subsignatus , Young, 1969: 2.
Bidessodes (Bidessodes) subsignatus , Young, 1986: 213; Biström, 1988: 7; Nilsson, 2016: 98.

#### Diagnosis.

This species lacks a carinate and spinous prosternum in either sex. The prosternal process is flat, the lateral margins are slightly convergent to the pointed apex. The male mesotibia is unmodified. The male metafemur and metatrochanter are unmodified (Fig. 91). The male abdominal ventrite VI is deeply indented laterally and apically impressed. The male median lobe in lateral aspect is slender and only slightly curved through most of its length to the apex which is abruptly curved and sharply pointed with a subapical tooth (Fig. 88). In ventral aspect the median lobe is deeply bifid with irregular lateral rami which terminate apically in laterally directed hooks (Fig. 89). The lateral lobe in lateral aspect has a small basal portion with the apical portion moderately large, and apically with a broad dorsally directed rounded lobe (Fig. 90). This is a small species, only about 2 mm in length, with maculate elytra (Fig. 87).

#### Distribution.

Known from Venezuela, Guyana and Brazil (Fig. 103).

### Bidessodes zimmermanni

Taxon classificationAnimaliaORDOFAMILIA

Hájek, 2012

Figs 92–96 , 103

Bidessus (Bidessodes) plicatus Zimmermann, 1921: 199; Blackwelder, 1944: 76; preoccupied, replaced by Bidessodes zimmermanni Hájek, 2012.
Bidessodes plicatus , Young, 1969: 2.
Bidessodes (Bidessodes) plicatus , Young, 1986: 218; Biström, 1988: 7.
Bidessodes zimmermanni Hájek, 2012: 67; Nilsson, 2016: 98; replacement for Bidessus plicatus Zimmermann, 1921.

#### Diagnosis.

This species lacks a carinate and spinous prosternum in males and females. The terminal visible abdominal ventrite is broadly impressed apically. The male mesofemur is apically somewhat swollen. The male mesotibia is basally bent. The male metatibia and metatrochanter are relatively unmodified (Fig. 96). The male median lobe in lateral aspect is evenly curved, somewhat expanded medially and apically narrowly rounded (Fig. 93). In ventral aspect the median lobe is narrow and apically simple with short setae (Fig. 94). The lateral lobe in lateral aspect has the basal segment short and irregularly sinuate, the apical portion is extremely large, elongate subtriangular and sinuate with the apex sharply pointed (Fig. 95).

#### Distribution.

This species is only known from Mato Grosso, Brazil (Fig. 103).

##### Checklist of species in *Bidessodes*

***Bidessodes* Régimbart, 1895**

*Bidessodes acharistus* Young, 1986

*Bidessodes charaxinus* Young, 1986

*Bidessodes chlorus* **sp. n.**

*Bidessodes demarcoi* Braga & Ferreira-Jr., 2009

*Bidessus elongatus* (Sharp, 1882) (*Bidessus*)

*Bidessodes erythros* **sp. n.**

*Bidessodes evanidus* Young, 1986

*Bidessodes fragilis* Régimbart, 1900

*Bidessodes franki* (Spangler, 1981) (*Youngulus*)

*Bidessodes hamadae* Braga & Ferreira-Jr., 2009

*Bidessodes hygrobius* Young, 1986

*Bidessodes jucundus* Young, 1986

*Bidessus knischi* (Zimmermann, 1921) (*Bidessus*)

=*Hughbosdinius leechi* Spangler, 1981

*Bidessodes leukus* **sp. n.**

*Bidessodes melas* **sp. n.**

*Bidessodes nessimiani* Braga & Ferreira-Jr., 2009

*Bidessus obscuripennis* (Zimmermann, 1921) (*Bidessus*)

*Bidessodes semistriatus* Régimbart, 1900

*Bidessus subsignatus* (Zimmermann, 1921) (*Bidessus*)

*Bidessodes zimmermanni* Hájek, 2012

=*Bidessus plicatus* Zimmermann, 1921

**Figures 97–98.**
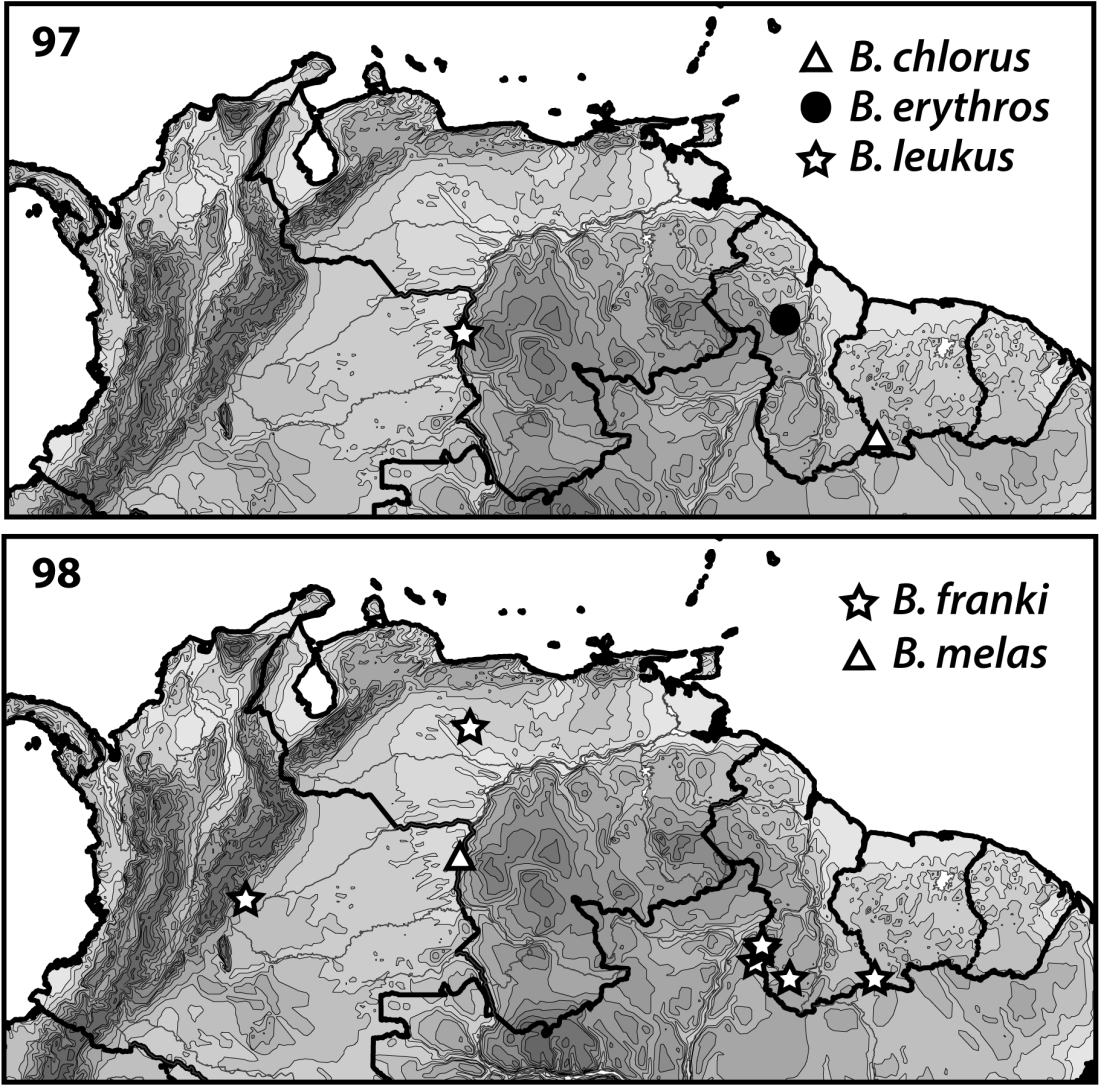
Distributions of *Bidessodes* species based on examined specimens and published records.

**Figures 99–100.**
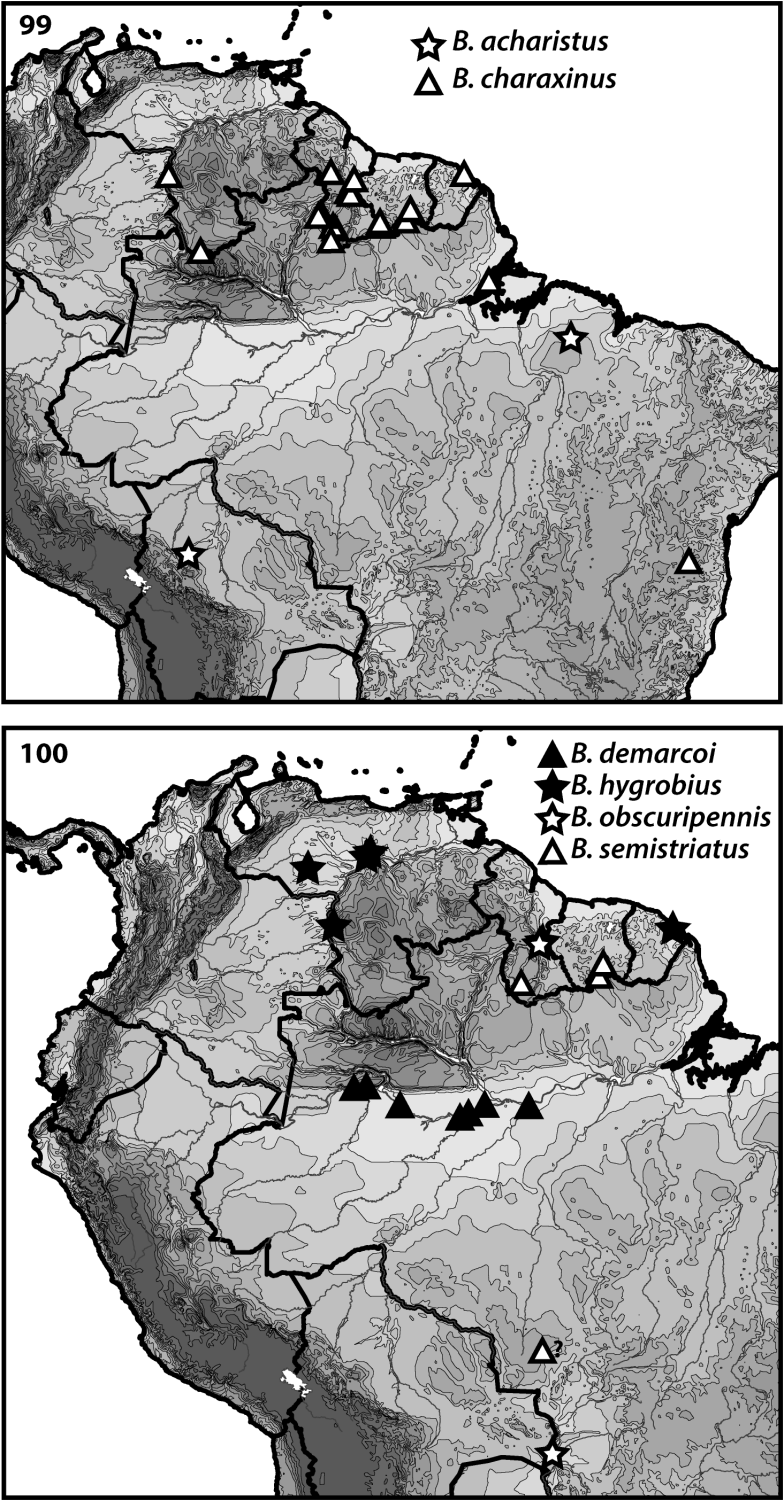
Distributions of *Bidessodes* species based on examined specimens and published records.

**Figures 101–102.**
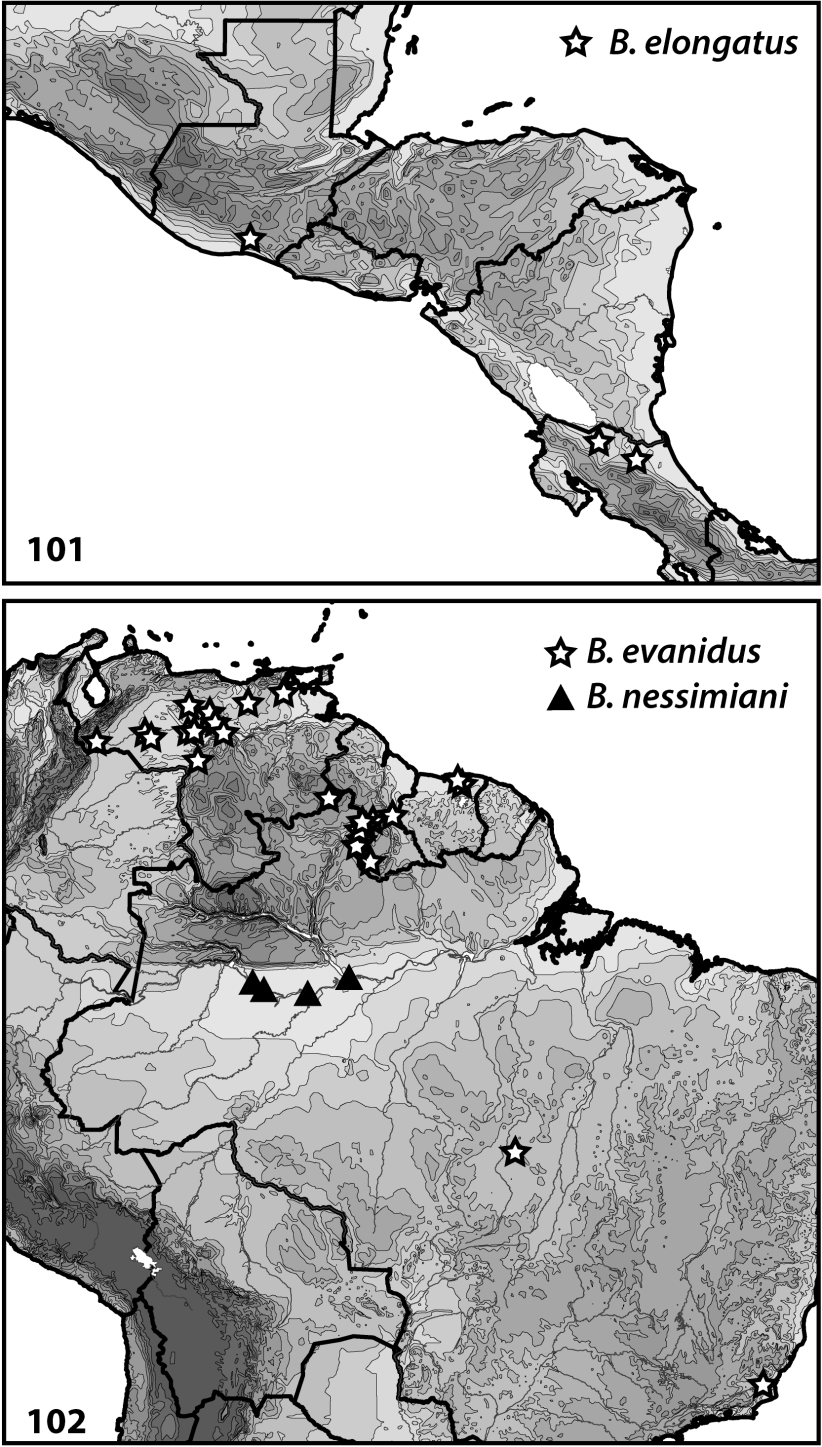
Distributions of *Bidessodes* species based on examined specimens and published records.

**Figures 103–104.**
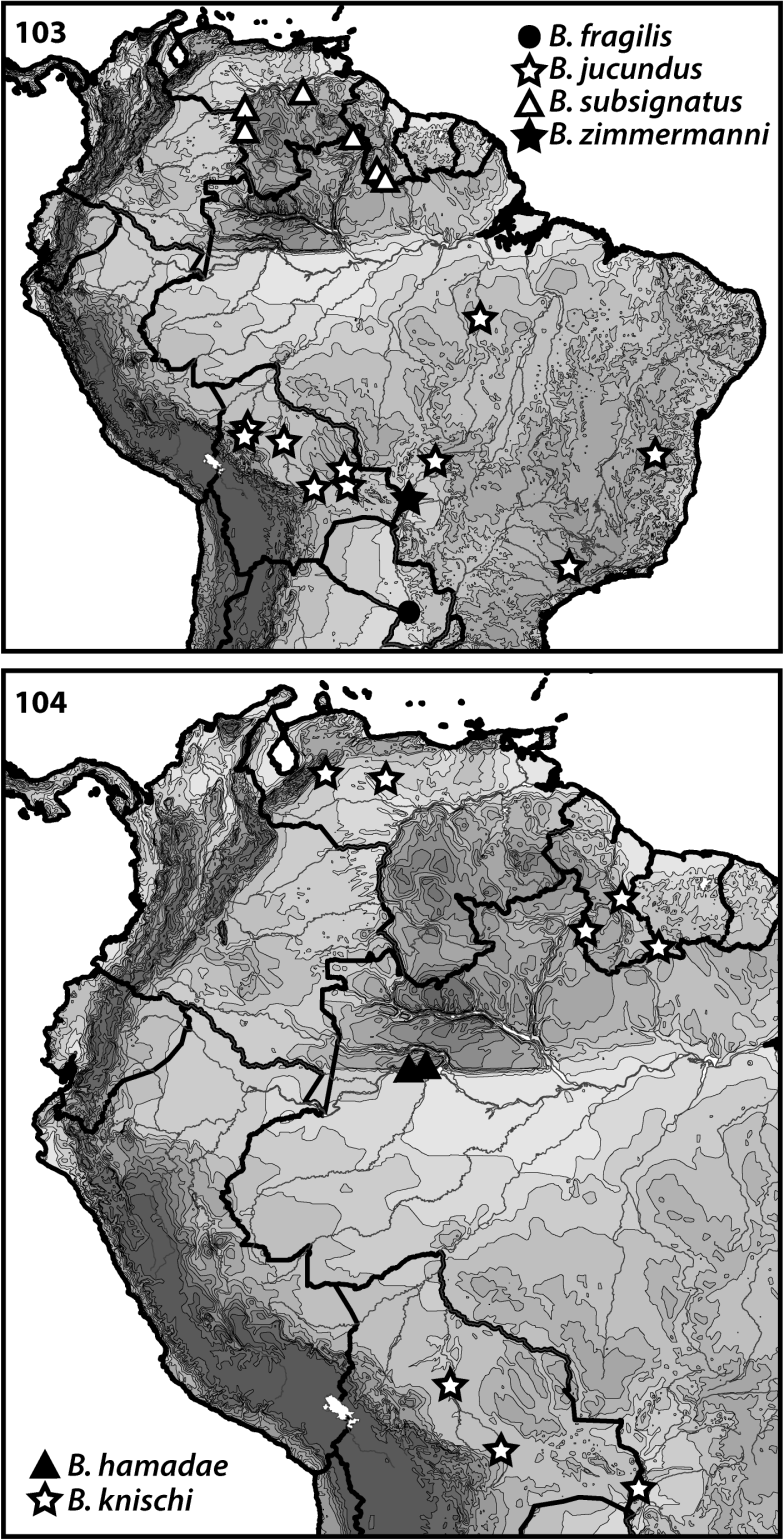
Distributions of *Bidessodes* species based on examined specimens and published records.

## Supplementary Material

XML Treatment for Bidessodes

XML Treatment for Bidessodes chlorus

XML Treatment for Bidessodes erythros

XML Treatment for Bidessodes leukus

XML Treatment for Bidessodes melas

XML Treatment for Bidessodes acharistus

XML Treatment for Bidessodes charaxinus

XML Treatment for Bidessodes demarcoi

XML Treatment for Bidessodes elongatus

XML Treatment for Bidessodes evanidus

XML Treatment for Bidessodes fragilis

XML Treatment for Bidessodes franki

XML Treatment for Bidessodes hamadae

XML Treatment for Bidessodes hygrobius

XML Treatment for Bidessodes jucundus

XML Treatment for Bidessodes knischi

XML Treatment for Bidessodes nessimiani

XML Treatment for Bidessodes obscuripennis

XML Treatment for Bidessodes semistriatus

XML Treatment for Bidessodes subsignatus

XML Treatment for Bidessodes zimmermanni
